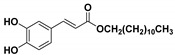# Modulation of ERK1/2 and Akt Pathways Involved in the Neurotrophic Action of Caffeic Acid Alkyl Esters

**DOI:** 10.3390/molecules23123340

**Published:** 2018-12-17

**Authors:** Razieh Hosseini, Fatemeh Moosavi, Tiago Silva, Hamid Rajaian, Seyed Younes Hosseini, Samaneh Bina, Luciano Saso, Ramin Miri, Fernanda Borges, Omidreza Firuzi

**Affiliations:** 1 Medicinal and Natural Products Chemistry Research Center, Shiraz University of Medical Sciences, Shiraz 71348-5373, Iran; hosseini_945@yahoo.com (R.H.); moosavi4891@yahoo.com (F.M.); sbina.biology@gmail.com (S.B.); Ramin.miri.15@gmail.com (R.M.);; 2 Department of Pharmacology, School of Veterinary Medicine, Shiraz University, Shiraz 71441-69155, Iran; hrajaian@yahoo.com; 3 CIQUP/Department of Chemistry and Biochemistry, Faculty of Sciences, University of Porto, 4169-007 Porto, Portugal; ntb.silva@gmail.com; 4 Department of Bacteriology and Virology, Shiraz University of Medical Sciences, Shiraz 71348-45794, Iran; hosseinisy22@yahoo.com; 5 Department of Physiology and Pharmacology “Vittorio Erspamer”, Sapienza University of Rome, 00185 Rome, Italy; luciano.saso@uniroma1.it

**Keywords:** hydroxycinnamic acids, neurodegenerative diseases, neurotrophic factors, polyphenols

## Abstract

Neurodegenerative diseases affect millions of human lives all over the world. The number of afflicted patients is rapidly growing, and disease-modifying agents are urgently needed. Caffeic acid, an important member of the hydroxycinnamic acid family of polyphenols, has considerable neurotrophic effects. We have previously shown how caffeate alkyl ester derivatives significantly promote survival and differentiation in neuronal cells. In this study, the mechanisms by which these ester derivatives exert their neurotrophic effects are examined. A series of eight caffeic acid esters with different alkyl chain lengths, ranging from methyl (CAF1) to dodecyl esters (CAF8), were synthesized and studied for their influence on neurotrophic signaling pathways. Caffeate esters did not induce tropomyosin-receptor kinase A (TrkA) phosphorylation, which was assessed by immunoblotting up to a concentration of 25 µM. NIH/3T3 cells overexpressing TrkA were generated to further examine phosphorylation of this receptor tyrosine kinase. None of the esters induced TrkA phosphorylation in these cells either. Assessment of the effect of caffeate derivatives on downstream neurotrophic pathways by immunoblotting showed that the most potent esters, decyl caffeate (CAF7) and dodecyl caffeate (CAF8) caused extracellular signal-regulated kinase (ERK1/2) and Akt serine threonine kinase phosphorylation in PC12 cells at 5 and 25 µM concentrations. In conclusion, this study shows that caffeate esters exert their neurotrophic action by modulation of ERK1/2 and Akt signaling pathways in neuronal cells, and further demonstrates the potential therapeutic implications of these derivatives for neurodegenerative diseases.

## 1. Introduction

Polyphenols are an important group of phytochemicals abundant in the human diet [1]. Polyphenols have been extensively studied for their antioxidant activities [2,3,4,5], and their possible role in the prevention of various pathologies such as cancer and cardiovascular disorders has been well documented [6]. Neuroprotective effects of polyphenols against various diseases of the nervous system have been addressed in a number of studies [7,8,9,10,11,12,13], and have been reported to be of therapeutic value for neurodegenerative diseases [14,15].

Hydroxycinnamic acids (HCAs) are an important subgroup of polyphenols which are widely distributed in plants and are abundant in fruits, vegetables, and beverages [16,17,18]. In particular, *p*-coumaric, caffeic, ferulic, and sinapic acids have been highlighted due to their relevant biological activities [9,19,20,21,22].

Neurotrophic factors or neurotrophins, such as nerve growth factor (NGF) and brain derived neurotrophic factor (BDNF), promote neuronal survival, proliferation, and differentiation. Efforts have recently been focused on neurotrophins’ therapeutic applications within neurodegenerative diseases, such as Alzheimer’s and Parkinson’s [23]. However, due to their poor pharmacokinetic profile, the scientific community has become increasingly interested in the search for small molecule agents that mimic the action of neurotrophins or potentiate their effects [24,25].

Tropomyosin-receptor kinases (Trk) are a family of receptor tyrosine kinases that mediate the biological effects of neurotrophins. Trk receptors, including NTRK1, NTRK2, and NTRK3 (TrkA, TrkB, and TrkC), affect neuronal survival and differentiation through several signaling cascades [26]. Activation and phosphorylation of Trk receptors upon ligand binding leads to the activation of Kirsten rat sarcoma proto-oncogene (Ras), phosphoinositide 3-kinase (PI3K), phospholipase C-γ1, and downstream signaling pathways such as extracellular signal-regulated kinase (ERK) and Akt [25].

In an earlier study we showed that alkyl esters of HCAs, and, in particular, caffeic acid ester derivatives, display relevant neuroprotective effects in serum-deprived conditions and promote neurite outgrowth in PC12 cells [27]. To gain insight into the neurotrophic mechanisms regulating the HCAs’ protective effects, the influence of some of the most potent derivatives on the phosphorylation of the TrkA receptor and its downstream signaling pathways (ERK1/2 and Akt) is examined in this study.

## 2. Results

### 2.1. Chemistry

All synthesized derivatives (as outlined in Table 1) were obtained in moderate to high yields and fully characterized by magnetic resonance spectroscopy (^1^H NMR, ^13^C NMR and DEPT135) and mass spectrometry [27].

### 2.2. Effect of K252a and Caffeic Acid Ester Derivatives on the Viability of Serum-Deprived PC12 Cells

**Figure 1:**
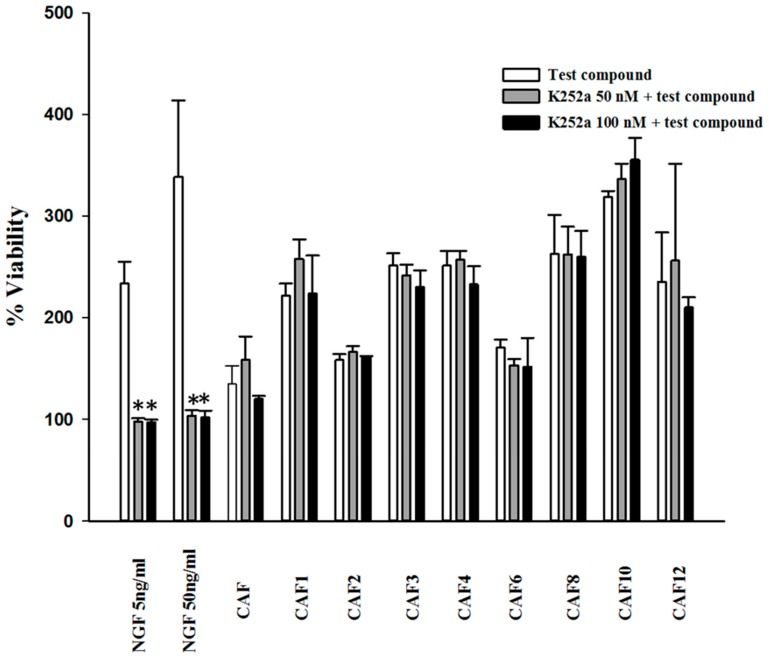
Effects of caffeic acid ester derivatives on the viability of serum-deprived PC12 cells in the presence of K252a. PC12 cells were seeded in 96-well plates deprived of serum and were treated with caffeate esters at 25 µM, either separately or concomitant with K252a, a TrkA antagonist, at 50 and 100 nM. Cell viability was measured after 48 h using a 3-[4,5 dimethylthiazol-2-yl]-2,5-diphenyl-tetrazolium bromide (MTT) assay. The results are plotted as the percentage of cell survival compared to untreated cells and expressed as the mean ± standard error of mean (SEM). While the presence of K252a completely blocked the effect of nerve growth factor (NGF), it did not alter the viability increase induced by caffeic acid ester derivatives. * Significantly different from cells treated solely with NGF (5 or 50 ng/mL) (*p* < 0.05).

Survival of serum-deprived PC12 cells exposed to K252a, a potent TrkA inhibitor, and caffeic acid ester derivatives, was evaluated using a 3-[4,5 dimethylthiazol-2-yl]-2,5-diphenyl-tetrazolium bromide (MTT) assay. As shown in Figure 1, K252a effectively antagonized the activity of NGF on promotion of neuronal cell survival in serum-deprived conditions. However, this inhibitor had no significant inhibitory effect on the protective activity of caffeate esters.

### 2.3. Assessment of TrkA Phosphorylation in PC12 Cells

**Figure 2:**
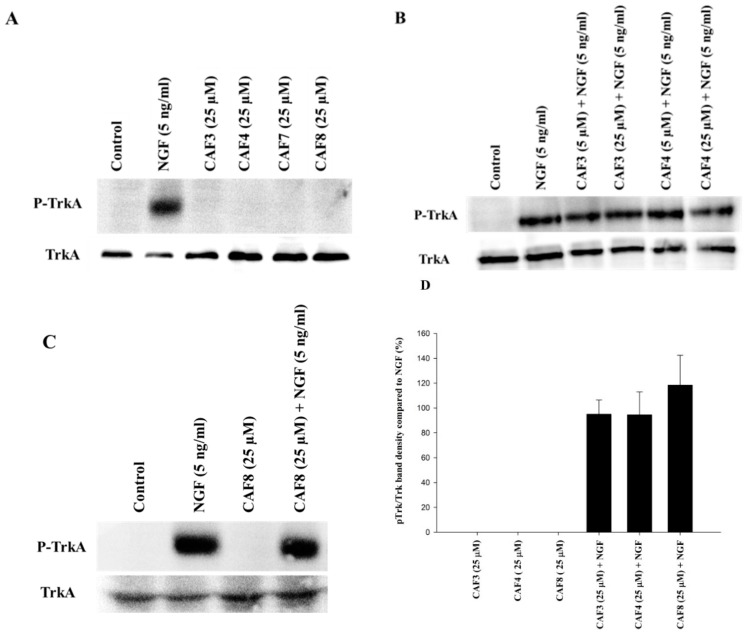
Assessment of tropomyosin-receptor kinase A (TrkA) phosphorylation in PC12 cells exposed to synthesized caffeate esters. PC12 cells were seeded in collagen-coated petri dishes and exposed to caffeate esters and/or NGF (5 ng/mL). After 30 min, the total cell protein was harvested in ice-cold RIPA lysis buffer containing protease inhibitors. The proteins were separated on 7.5% SDS−PAGE, transferred onto a PVDF membrane and probed with specific antibodies. No band was detected in cells treated with caffeate esters alone (**A**–**C**). The band intensities of p-TrkA and TrkA in PC12 cells treated with caffeate esters were quantified. The intensity of each p-TrkA band was first divided by the band intensity of the respective TrkA. The p-TrkA/TrkA ratio of each blot was then normalized to a p-TrkA/TrkA ratio of NGF at 5 ng/mL. This figure shows the mean of three experiments ± SEM. No significant effect was observed in cells treated with a combination of caffeate esters and NGF compared to cells treated only with NGF (**D**).

PC12 cells were exposed to synthesized compounds separately or in addition to NGF (5 ng/mL) for 10, 30, and 60 min periods. The total cell protein was then extracted and subjected to immunoblotting. At 30 and 60 min the maximum level of TrkA phosphorylation was observed and 30 min was chosen for comparison with the effect of caffeate esters. As shown in Figure 2A–C, NGF at 5 ng/mL noticeably induced a robust phosphorylation in TrkA. However, caffeate esters tested at 5 and 25 µM (only 25 µM data are shown) did not induce detectable TrkA phosphorylation. The concomitant incubation of synthesized derivatives with NGF did not significantly alter the level of phosphorylation in TrkA either (Figure 2B,C).

### 2.4. Establishment of NIH/3T3-TrkA Cells

**Figure 3:**
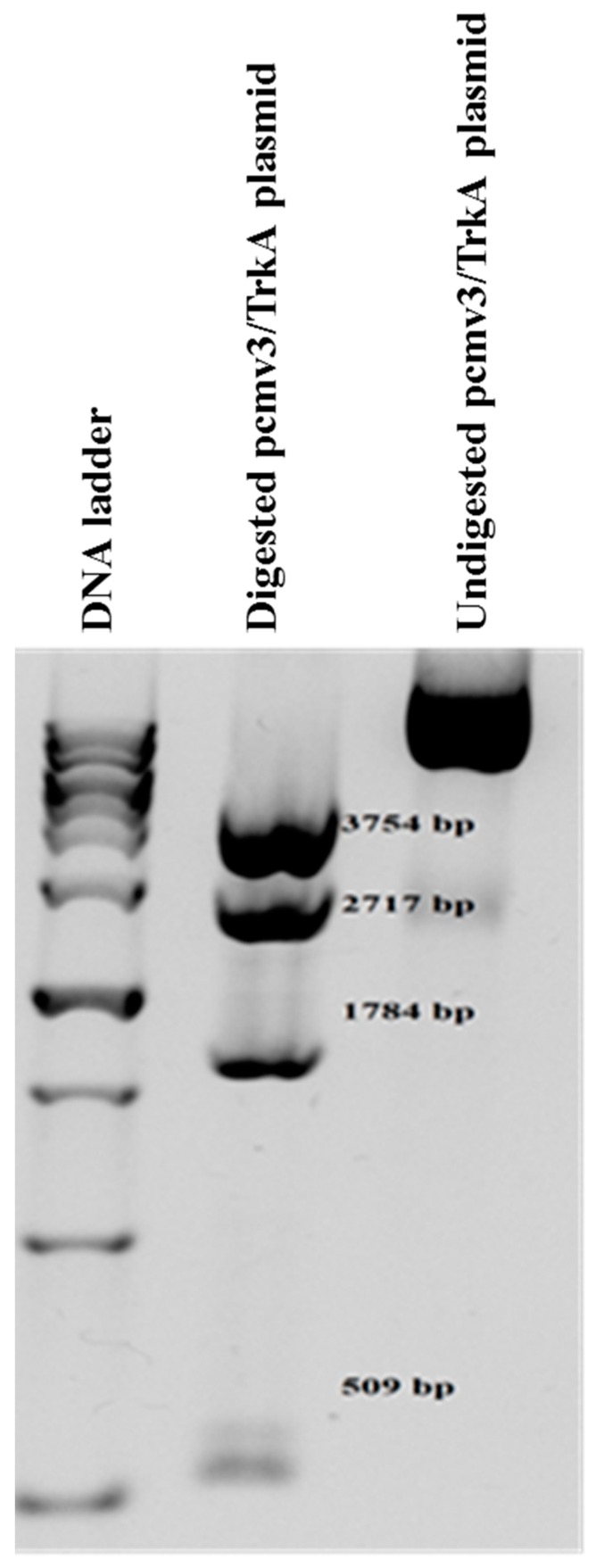
Restriction analysis of the pCMV3-TrkA expressing vector. The human pCMV3/TrkA plasmid was digested by *Nco* I and was then analyzed by gel electrophoresis. Four bands (509, 1784, 2717, and 3754 base pair fragments) were detected as indicators of plasmid integrity.

The restriction analysis of the pCMV3-TrkA expressing plasmid by restriction enzyme *Nco* I on agarose gel represented a pattern of four correct fragments (509, 1784, 2717 and 3754 base pair bands) (Figure 3), ensuring the integrity of the plasmid. The digestion of plasmid by *Pci* I also showed a linear mono-band pattern of expected size for the linear plasmid (near 9 Kb). The addition of Hygromycin B in culture media induced a significant cytotoxicity in the majority of cells which did not integrate the linear plasmid. However, the cells that retained the plasmid formed the growing cell colonies by this time in the presence of a toxic concentration (800 ug/mL) of Hygromycin B. Microscopic results also showed that NIH/3T3 cells were stably transfected with a linear-plasmid-containing human TrkA expressing cassette, as different colonies were growing suitably from a small number of passaged cells in the presence of Hygromycin B. Further functional protein assays also indicated the specific and correct expression of the TrkA protein, as is described later.

### 2.5. Assessment of TrkA Phosphorylation in NIH/3T3-TrkA Cells

**Figure 4:**
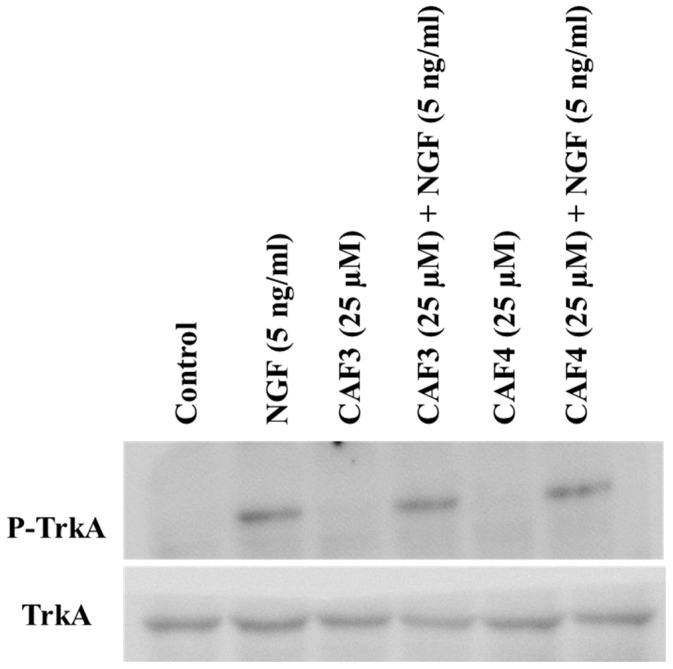
Assessment of TrkA phosphorylation in NIH/3T3-TrkA cells exposed to synthesized caffeate esters. NIH/3T3-TrkA cells were seeded in 12-well plates in Dulbecco’s modified Eagle’s medium (DMEM) containing 10% fetal bovine serum (FBS) for 48 h, then shifted to a low serum medium (2% FBS) for 24 h prior to exposure to hydroxycinnamic acid (HCA) alkyl esters (5 and 25 µM) and/or NGF (50 and 5 ng/mL) for 30 min. Cells were washed with phosphate buffer saline (PBS) and the proteins extracted in ice-cold RIPA lysis buffer containing protease inhibitors. The proteins were separated on 7.5% SDS−PAGE, transferred onto a PVDF membrane, and probed with specific antibodies. No band was detected in samples treated with caffeate esters alone.

The effect of caffeate derivatives was also evaluated in NIH/3T3 cells stably transfected with TrkA. These cells stably expressed high levels of TrkA and this overexpressed receptor was functional, since NGF (5 ng/mL) exposure led to its phosphorylation. However, as with the findings for PC12 cells, the caffeate esters did not show any effect on TrkA phosphorylation in these cells, neither by themselves nor in combination with NGF (Figure 4).

### 2.6. Assessment of ERK1/2 and Akt Phosphorylation in PC12 Cells

**Figure 5:**
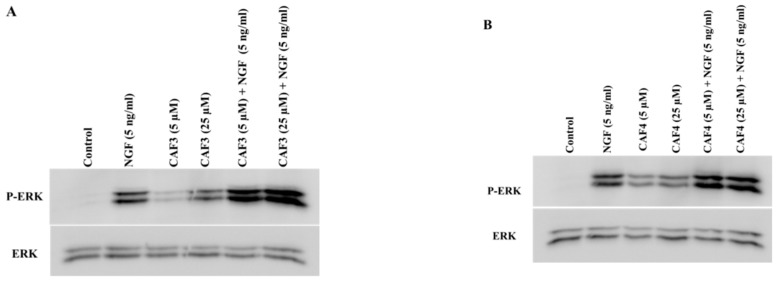

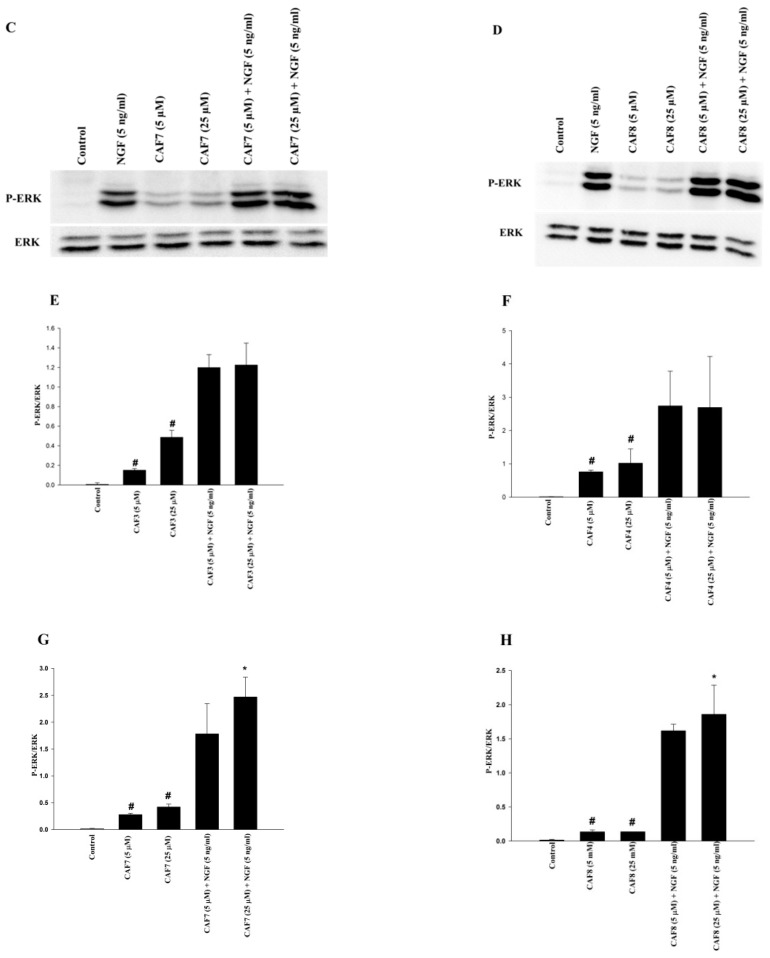
Assessment of extracellular signal-regulated kinase (ERK1/2) phosphorylation in PC12 cells treated with synthesized caffeate esters. PC12 cells were seeded in collagen-coated petri dishes and were exposed to caffeate esters and/or NGF (5 ng/mL). After 120 min, the total cell protein was harvested, separated by 7.5% SDS−PAGE, transferred onto a PVDF membrane and probed with specific antibodies (**A**–**D**). The band intensities of p-ERK1/2 and ERK were quantified. The intensity of each p-ERK1/2 band was first divided by the band intensity of the respective ERK. The p-ERK/ERK ratio in drug-treated cells was normalized to this ratio in NGF-treated cells (**E**–**H**). The figures show the mean of three experiments ± SEM. **^#^** Significantly different from untreated control cells (*p* < 0.05). * Significantly different from cells solely treated with NGF (5 ng/mL) (*p* < 0.05).

**Figure 6:**
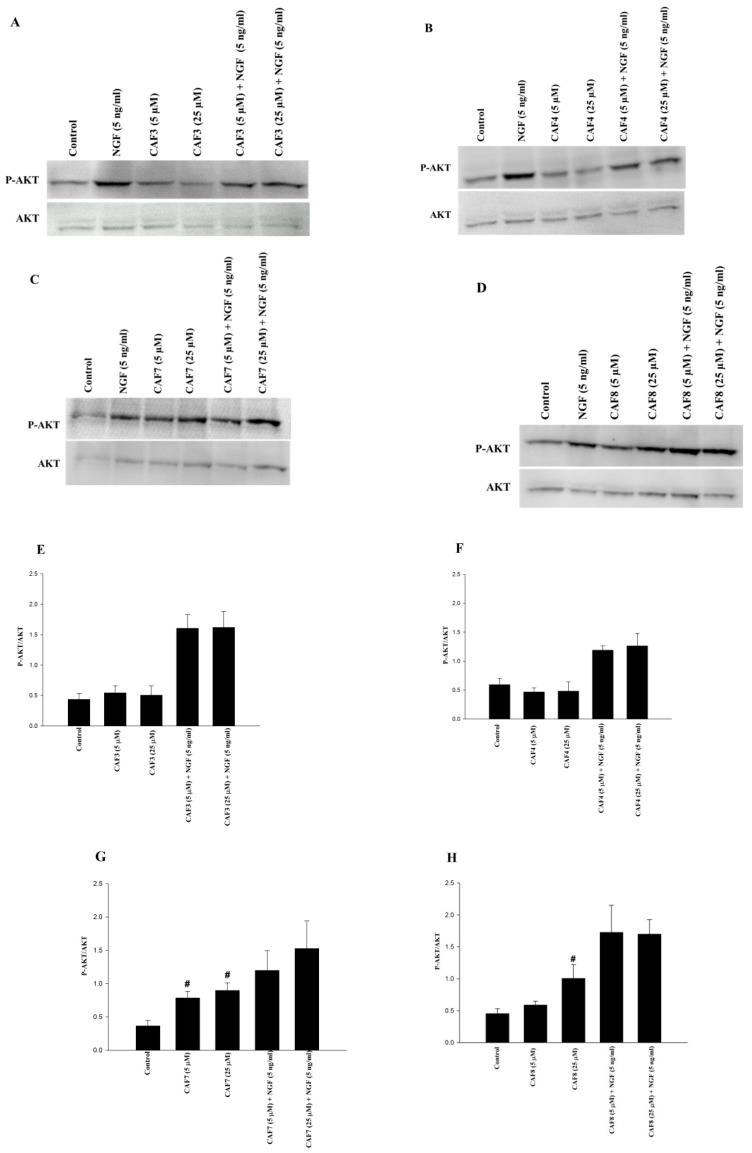
Assessment of AKT phosphorylation in PC12 cells treated with synthesized caffeate esters. PC12 cells were seeded in collagen-coated petri dishes and exposed to caffeate esters and/or NGF (5 ng/mL). After 120 min, the total cell protein was harvested, separated by 7.5% SDS−PAGE, transferred onto a PVDF membrane and probed with specific antibodies (**A**–**D**). The band intensities of p-AKT and AKT were quantified. The intensity of each p-AKT band was first divided by the band intensity of the respective AKT. The p-AKT/AKT ratio in drug-treated cells was then normalized to this ratio in NGF-treated cells (**E**–**H**). The figures show the mean of four experiments ± SEM. **^#^** Significantly different from untreated control cells (*p* < 0.05).

PC12 cells were exposed to synthesized compounds separately or together with NGF (5 ng/mL) for 120 min. The total cell proteins were then extracted and subjected to immunoblotting. CAF3, CAF4, CAF7, and CAF8 were tested at 5 and 25 µM. As shown in Figure 5 and Figure 6, NGF at 5 ng/mL noticeably induced phosphorylation in ERK1/2 and Akt. CAF3, CAF4, CAF7 and CAF8 induced detectable ERK1/2 phosphorylation in comparison to the control group (Figure 5A–D).

The concomitant incubation of CAF3 and CAF4 with NGF increased ERK1/2 phosphorylation (producing 1.2/1.2 and 2.7/2.6 folds, respectively, at 5/25 µM), although this enhancement did not reach statistical significance (Figure 5E,F). On the other hand, incubation of CAF7 and CAF8 at 25 µM with NGF at 5 ng/mL significantly increased ERK1/2 phosphorylation, compared to when the cells were simply exposed to NGF (Figure 5G,H).

As shown in Figure 6A–D, CAF3 and CAF4 did not induce detectable Akt phosphorylation at 5 and 25 µM when compared with the control, but CAF7 (at 5 and 25 µM) and CAF8 (at 25 µM) significantly induced Akt phosphorylation. The concomitant incubation of CAF3, CAF4, CAF7, and CAF8 with NGF (5 ng/mL) increased Akt phosphorylation (producing 1.6/1.6, 1.2/1.3, 1.2/1.5, and 1.7/1.7 folds, respectively, at 5/25 µM); these changes were not statistically significant, however (Figure 6E–H).

## 3. Discussion

In this study, the molecular mechanisms involved in the neurotrophic action of caffeic acid and seven caffeate alkyl esters on neurotrophic signaling pathways (including TrkA, ERK1/2 and Akt) in PC12 cells were examined. Our findings demonstrate that the neurotrophic activity of ester derivatives is brought about by the activation of ERK1/2 and Akt pathways.

Neurotrophins such as NGF and BDNF promote neuronal survival and protect neurons against various types of cell damage, and play important roles in cognition and memory [28,29,30,31,32,33]. Neurotrophins exhibit their function by activation of Trk receptors [34] and consequently the mitogen-activated protein kinase (MAPK) and PI3K/Akt pathways [35,36]. It is well established that activation of these signaling pathways plays a crucial role in some physiological functions of neurons, such as survival, proliferation, differentiation, and regulation of response to various growth factors [37,38,39,40,41], and ultimately may be of potential therapeutic significance for neurodegenerative diseases [42,43,44]. Since some small molecules are known to induce the activation of ERK1/2 and PI3K/Akt pathways [45,46], it is of therapeutic interest to use these synthetic or natural small molecule drugs to mimic the actions of NGF on ERK1/2 and PI3K/Akt pathways and neurite extension.

In a previous study, we indicated the significant effect of caffeate esters on the promotion of neuronal survival and enhancement of NGF-induced neurite outgrowth in PC12 cells [27]. In this study, in order to examine if the survival-enhancing effect of caffeate esters is induced by direct agonistic action on TrkA receptors, we examined the effect of the derivatives in the presence of K252a, a potent TrkA inhibitor. It was observed that although the TrkA inhibitor could completely block the survival enhancement effect of NGF at 5 and 50 ng/mL, it had no influence on the activity of caffeate esters. We confirmed this finding by using immunoblotting, which showed the caffeate esters with neurotrophic activity did not cause TrkA phosphorylation. Even when caffeate esters were applied together with NGF, they did not increase TrkA phosphorylation induced by NGF.

We wanted to confirm that this lack of direct activity on TrkA receptors is not a cell specific phenomenon and hence generated a cell line that stably overexpressed TrkA (NIH/3T3-TrkA). In accordance with the findings for PC12 cells we also confirmed that even in NIH/3T3-TrkA cells the caffeate esters did not cause TrkA phosphorylation. Similarly to what was observed for PC12 cells, even when caffeate esters were added in addition to NGF, they did not increase NGF-induced TrkA phosphorylation.

These observations clearly demonstrate that the action of caffeate esters is not directly mediated via agonistic action on TrkA receptors but probably by modulation of more downstream neurotrophic signaling pathways. Indeed, aside from a few phenolic compounds such as 7,8-dihydroxyflavone that exert direct agonistic action on TrkB receptors [47,48,49], most other phenolic compounds exert their neurotrophic effect on more downstream signaling pathways [13].

For this reason, we tested the activation of ERK1/2 and Akt signaling pathways via western blot. Our results indicate the significant capacity of caffeate ester derivatives (CAF3, CAF4, CAF7, and CAF8) to induce phosphorylation of ERK1/2 at 5 and 25 µM concentrations. Other research has demonstrated activation of the ERK1/2 signaling pathway by HCA derivatives and other polyphenols [13,50]. For example, the flavonoid luteolin protects PC12 cells via activation of ERK1/2. This compound also increases neurite outgrowth and expression of the protein GAP-43, effects that can be blocked by ERK1/2 inhibition [51]. Carnosic acid and rosmarinic acid, which may be isolated from the *Rosmarinus officinalis* plant, also exhibit neurotrophic effects in PC12 cells via the ERK1/2 signaling pathway [52].

Since the activation of the Akt pathway is well known to play an important role in neuronal survival, we also examined the effects of active caffeate ester derivatives on this signaling pathway. CAF3 and CAF4 did not have significant effects on Akt phosphorylation, but CAF7 at 5 and 25 µM, and CAF8 at 25 µM, significantly induced Akt phosphorylation. In our previous study, the findings of the docking examination clearly showed that caffeate esters had reasonable interactions with the active site of PI3K/Akt, as the potential molecular target and elongation of alkyl side chains in these esters resulted in enhanced binding interaction with the PI3K active site [27].

It has been previously reported that methyl 3,4-dihydroxybenzoate, a phenolic acid derivative, promotes neuronal survival and neurite outgrowth via activation of the PI3K/Akt signaling pathway in cultured primary cortical neurons, and that these effects can be inhibited by a PI3K-specific inhibitor [53]. Moreover, caffeoylserotonin has been shown to have protective effects against oxidative stress-induced cell death in keratinocytes via the activation of the PI3K/Akt pathway and subsequent activation of nuclear factor erythroid 2–related factor 2 (Nrf2) [54]. In another study, scutellarin, a polyphenol containing a catechol moiety and a caffeic acid ester fraction (a mixture of different esters of caffeic acid, mainly consisting of quinic acid esters) has shown protective effects within primary rat astrocytes against hypoxia through the production and release of neurotrophins by stimulation of cAMP response element-binding protein (CREB) and Akt signaling pathways [55]. Protective effects of caffeic acid and caffeic acid phenethyl ester against acrolein-induced neurotoxicity in mouse hippocampal neuronal cells has also been shown to be mediated by MAPKs and Akt signaling pathways [56].

The lipophilic structure of caffeic acid phenetyl ester (CAPE) has the capacity to cross the blood brain barrier (BBB), and several reports have shown the therapeutic potential of this caffeic acid derivative in neurodegenerative diseases and other CNS disorders in vivo [57,58]. The effect of CAPE on dopaminergic neuronal loss induced by 6-hydroxydopamine in rats is also consistent with previous evidence indicating the ability of this compound to cross the BBB [59]. Studies in mice have also shown that salvianolic acid A, a caffeic acid derivative, has protective properties against ischemic brain injury, which similarly reveals the ability of this compound to cross the BBB [60].

## 4. Materials and Methods

### 4.1. Reagents

Dimethyl sulfoxide, absolute ethanol and methanol were obtained from Merck (Darmstadt, Germany). 3-(4,5-Dimethylthiazol-2-yl)-2,5-diphenyl tetrazolium bromide (MTT), rat nerve growth factor (NGF-β) and all other reagents needed for chemical synthesis were obtained from Sigma-Aldrich (St Louis, MO, USA). Dulbecco’s modified Eagle’s medium (DMEM), fetal bovine serum (FBS), and horse serum were acquired from Invitrogen (San Diego, CA, USA), while penicillin/streptomycin, RPMI 1640, sterile phosphate-buffered saline (PBS), and trypsin EDTA 0.25% were purchased from Biosera (Ringmer, UK). Anti-phospho-TrkA, anti-MAP kinase ERK1/2, anti-phospho-MAP kinase ERK1/2 (pThr202/Tyr204), and anti-phospho-Akt antibodies were obtained from Cell Signaling (Danvers, MA, USA). Anti-TrkA, anti-Akt antibodies, and K252a were obtained from Santa Cruz Biotechnology (Santa Cruz, CA, USA). The *Homo sapiens* TrkA/NTRK1 cDNA clone-containing plasmid was purchased from Sino Biologicals (Beijing, China). A plasmid extraction Miniprep kit was obtained from Invitek Inc. (Berlin, Germany). Deionized water was used in all experiments.

### 4.2. Chemistry

All compounds (as outlined in Table 1) were synthesized as previously described [27]. Their structural data is in accordance with the literature [27].

### 4.3. Cell Culture

PC12 cells (rat pheochromocytoma) were generously gifted by Professor Lloyd A. Greene (Department of Pathology and Cell Biology, Columbia University, New York, NY, USA). PC12 cells were grown in a RPMI 1640 medium, supplemented with 10% heat-inactivated horse serum, 5% heat-inactivated fetal bovine serum, 100 U/mL penicillin G, and 100 mg/mL streptomycin. They were cultured on collagen-coated plates and incubated at 37 °C in humidified air containing 5% CO_2_ [61]. Two-thirds of the growing medium was changed every two to three days and the cells were sub-cultured once a week. Trypsin 0.25% was used for detachment of cells.

### 4.4. Cell Viability Assay Using K252a

The cells were seeded in collagen coated 96-well microplates at a density of 6 × 10^5^ cells/mL (80 µL per well) in a serum-free medium and treated with caffeic acid ester derivatives at 25 µM either separately or in combination with K252a (50 and 100 nM). NGF, at concentrations of 5 and 50 ng/mL, was used as the positive control. Test compounds were first dissolved in DMSO, and then diluted in growth medium at a final concentration of 25 µM and added to 96-well plates in triplicate. The final concentration of DMSO did not exceed 0.25% in each well. This concentration had no effect on cell viability. After incubation of cells at 37 °C for 48 h, the media were removed from the wells and 20 μL aliquots of MTT solution (0.5 mg/mL) were added to each well, followed by incubation for another 90 min. Formazan crystals were dissolved in DMSO (200 μL/well) and incubated at 37 °C for 60 min. The plates were then shaken for another 30 min. The absorbance was measured with a microplate reader (Model 680, Bio-Rad, Hercules, CA, USA) at 570 nm with a background correction of 650 nm.

### 4.5. Immunoblotting

PC12 cells (6 × 10^5^ cells/mL) were seeded in collagen-coated 35 × 10 mm dishes in a normal growth medium for 48 h. They were then shifted to a low serum medium (2% horse serum and 1% FBS) and after 24 h, caffeic acid ester derivatives (5 and 25 µM) and/or NGF (50 and 5 ng/mL) were added. After 10, 30, 60, and 120 min, the cells were washed with phosphate buffer saline, scraped in ice-cold RIPA lysis buffer (20 mM Tris, 1 mM EDTA, 150 mM NaCl, 5% sodium deoxycholate, 1% NP-40, and 0.1% SDS) containing 1 μM phenylmethyl sulfonyl fluoride, 1 μM NaF, 10 mM sodium pyrophosphate, 2 mM sodium orthovanadate, and a protease inhibitor cocktail (Roche, Germany), and incubated on ice for 15 min. Cellular debris was removed by centrifugation (12,000 g for 20 min) at 4 °C and the cell lysate was carefully transferred to micro-tubes.

The cell lysate (25 μg) was separated on 7.5% SDS−PAGE and transferred onto a PVDF membrane (PerkinElmer, Waltham, MA, USA) at 60 V for 3 h. The membranes were incubated in a blocking buffer (4% BSA in TBS containing 0.1% Tween-20) for 50 min. Blots were incubated at 4 °C with primary antibodies for 24 h. After three washes with TBST, the blots were incubated with appropriate horseradish peroxidase-conjugated secondary antibodies (1:1000) at room temperature (Santa Cruz Biotechnology, Santa Cruz, CA, USA) for 1 h. The blots were washed with TBST, and the proteins detected by enhanced chemiluminescence (ECL) (GE Healthcare, Buckinghamshire, UK). Images were obtained with a G: Box Chemi-XR5 GeneSys image analyzer. The intensities of bands were calculated with the software GeneTools (SyneGene, Cambridge, UK) for Windows.

### 4.6. Establishment of Stable TrkA Overexpressing NIH/3T3 Cells

NIH/3T3 cells were used to generate TrkA-overexpressing cells. These have low levels of endogenous TrkA and are a common choice for transfection host cells. They have been frequently used for assessment of the biological processes and roles of several different target genes involved in the pathogenesis of neurodegenerative diseases [62,63,64].

The pCMV3/TrkA plasmid containing a TrkA coding sequence was transformed into a XL-1 blue competent bacteria. The plasmids of truly transformed colonies were extracted with a plasmid DNA extraction Miniprep kit according to the manufacturer’s instructions (Invitek Inc., Berlin, Germany).

Restriction digestion analysis with *Nco* I was done to evaluate the integrity of the amplified plasmid. Following this, digestion by *Pci* I was performed to produce a linear form of the plasmid before transfection. The enzyme was selected because of its single recognition site among vectors, which is located outside the TrkA expressing cassette. The linearized vector was then extracted using a gel extraction kit (MN Inc., Duren, Germany), and quantified using a NanoDrop instrument. NIH/3T3 cells were cultured in DMEM supplemented with 10% FBS, 100 IU/mL penicillin G, and 100 mg/mL streptomycin, and then maintained at 37 °C in a humidified atmosphere with 5% CO_2_ and 95% humidity. Linear plasmid DNA transfection in NIH/3T3 cells was performed in the following manner. The cells were cultured in 6-well plates with a density of 20,000 cells/well, and incubated for 24 h. Afterwards, cells were transfected with a linearized pCMV3-TrkA vector using a Lipofectamine-2000^TM^ transfection reagent according to the manufacturer’s instructions. Briefly, a mixture of 1.5 μL Lipofectamine and 800 ng of linear plasmid were added to cells in a serum-free medium. After 3 h the medium was replaced with a complete 10% culture DMEM medium. Following an additional 24 h of incubation, the cells were treated with hygromycin B (Sigma Aldrich Inco., St. Louis, MO, USA) of a final concentration of 800 µg/mL. Then, due to the half-life of the antibiotic, and to get rid of dead cells, the old media were removed and the cells were fed with fresh media containing hygromycin B (of a final concentration of 800 µg/mL) two more times with 2-day intervals in between. When cell colonies appeared to be growing, they were harvested (on day six). For further selection of suitable cell colonies that received the vector, the cells were sub-cultured in 48-wells plate at a density of 5,000 cells/mL and the hygromycin B (of a concentration of 800 µg/mL) selection process repeated for another six days, in the same way as before. On day six, different cells colonies were finally harvested and introduced into functional analysis.

### 4.7. Statistical Analysis

Values are expressed as the mean ± SEM of independent experiments. Statistical analyses were carried out using analysis of variance (ANOVA) followed by appropriate post hoc tests including multiple comparison tests (LSD). All statistical analyses were performed using an SPSS statistical software package Version 13.1 for Windows (SPSS Inc., Chicago, IL, USA), and a probability value of less than 0.05 was accepted as statistically significant.

## 5. Conclusions

In conclusion, this study is the first to report the mechanism of neurotrophic action of alkyl ester derivatives of caffeic acid. It has found that activation of TrkA receptors is not involved in the mechanism of the action of these derivatives. However, the phosphorylation of downstream signaling pathways of TrkA receptors, such as ERK1/2 and Akt, probably plays a critical role in mediating neurotrophic effects of the compounds in promoting cell survival and induction of neurite outgrowth in neuronal cells. These phenolic compounds capable of modulating crucial neurotrophic signaling pathways represent promising agents for the discovery of neurotrophic agents of potential therapeutic use for neurodegenerative diseases.

## Figures and Tables

**Table 1:** Chemical structures of caffeic acid ester derivatives used in this study.

Compound	Structure
**CAF1: C1**	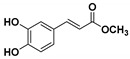
**CAF2: C2**	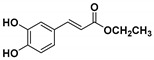
**CAF3: C3**	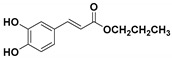
**CAF4: C4**	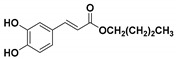
**CAF5: C6**	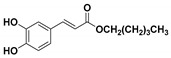
**CAF6: C8**	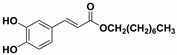
**CAF7: C10**	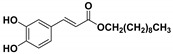
**CAF8: C12**